# From genes to trajectories: mapping genetic influences on Huntington’s disease progression

**DOI:** 10.1093/bioinformatics/btag072

**Published:** 2026-02-15

**Authors:** Sanjoy Dey, Zhaonan Sun, John Warner, Eileen Koski, Elif Eyigoz, Swati Sathe, Cristina Sampaio, Jianying Hu

**Affiliations:** IBM Research, Yorktown Heights, NY 10598, United States; IBM Research, Yorktown Heights, NY 10598, United States; CHDI Management, CHDI Foundation, Princeton, NJ 08540, United States; IBM Research, Yorktown Heights, NY 10598, United States; IBM Research, Yorktown Heights, NY 10598, United States; CHDI Management, CHDI Foundation, Princeton, NJ 08540, United States; CHDI Management, CHDI Foundation, Princeton, NJ 08540, United States; IBM Research, Yorktown Heights, NY 10598, United States

## Abstract

**Motivation:**

There are many diseases with established genetic factors, such as Huntington’s disease (HD), that are characterized by variable rates of progression. However, beyond the contribution of the known genetic factors - in this case the Huntingtin (HTT) gene - the impact of the full human genome on the natural progression of such diseases throughout a patient’s life remains largely unknown. The increased availability of genome wide association (GWA) data in HD gene expansion carriers (HDGECs), combined with the clinical assessment scores on the same set of patients, has provided a perfect opportunity to assess the potentially broader genetic impact on the natural progression of HD.

**Results:**

We present a genetics-driven, probabilistic disease progression model designed to identify and investigate the ways in which a range of genetic factors affect the natural progression of HD. When applied to a clinico-genomic HD dataset, our model identified several single nucleotide polymorphisms (SNPs) with previously unreported effects on disease progression that act at distinct stages and with varying magnitudes. This discovery may shed light on the potential mechanistic impact of previously unidentified genes on HD that may have implications for clinical management. As increasing amounts of GWA data become available more generally, we anticipate that this modeling framework will be broadly applicable to other diseases with strong genetic components.

**Availability and implementation:**

The source code for IHDPM is available at https://github.com/BiomedSciAI/IHDPM

## 1 Introduction

A 2020 study of data in the ORPHANET database identified over 5000 known genetic disorders (Pavan *et al.* 2017, Saboo *et al.* 2021), many caused by known point mutations, however the impact of the full human genome on disease onset and progression requires data that is not yet widely available. In the case of Huntington’s disease [HD], the increased availability of genome wide association (GWA) data in HD gene expansion carriers (HDGECs), combined with the clinical assessment scores on the same set of patients, has provided a perfect opportunity to assess the potentially broader impact of the full human genome on the natural progression of HD.

Huntington’s Disease (HD) is an autosomal dominant inherited disorder that results in neuro-degenerative effects caused by an unstable expansion in a trinucleotide (CAG) repeat in the huntingtin (HTT) gene. HD is characterized by progressive motor, cognitive/behavioral, and functional symptoms of a person who carries the disease carrier gene CAG (Ross *et al.* 2014). There is currently no known cure for the disease itself, but better understanding of natural progression of HD is crucial for early detection and implementation of potential disease modifying interventions. Development of *Disease Progression Modeling* (DPM) (Mould 2012) techniques that track severity of diseases over time, is critical in helping clinicians and researchers develop such understanding. Large longitudinal histories from patients collected in observational databases such as disease registries (Gliklich *et al.* 2005) and Electronic Health Records (EHRs) (Jensen *et al.* 2012) provide rich structured sources of information to enable data-driven approaches.

In recent years, several large-scale observational studies of HD have been conducted to examine the triad of clinical measures for motor, cognitive/behavioral, and functional symptoms with the hope of better understanding the natural history and pathophysiology of disease (Sun *et al.* 2019). In a previous study, an Integrated HD Progression Model (IHDPM) (Sun *et al.* 2019) was developed that combines multi-dimensional clinical measures and is trained on aggregate clinical measure data from multiple observational studies to track the natural progression of HD. However, disease progression is rarely homogeneous across patients.

Although HD is known to be caused by unstable expansion in a trinucleotide (CAG) repeat in the huntingtin (HTT) gene (MacDonald *et al.* 1993, Ross *et al.* 2014), other genes are known to modify this effect. The precise nature of the effects of these modifier genes on the natural progression of the disease throughout a patient’s life is a matter of current scientific interest (Lee *et al.* 2019). To investigate further, genome wide association (GWA) data have been collected in HD gene expansion carriers (HDGECs) for a subset of the same cohort in this study. Moreover, the natural progression of HD may vary across patients depending on their genetic makeup. Therefore, discovering diverse progression pathways of HD may help identify patients with higher risk for HD in early stages, which can ultimately enhance the development of precision medicine. In this work, we extended the IHDPM using Continuous time Hidden Markov Models (CTHMM) to examine whether genetic variations identified from genome-wide associations (GWA) have an impact on the natural progression of HD at different stages characterized by changes in the clinical observational data.

Continuous time Hidden Markov Models (CTHMM) are statistical models that allow both transitions between disease stages and observations to occur at arbitrary time instances (Cox and Miller 1965, Jackson 2011). They provide a flexible framework for modeling irregularly sampled longitudinal data from disease registry or EHRs. In the CTHMM framework, the progression of a target disease is segmented into *M* states and completely characterized by the duration of states and transitions between them. The state and transition parameters are shared by all patients, and their longitudinal observations are assigned to one of *M* states, thus aligning the incomplete trajectories of different patients in the cohort.

While CTHMMs elegantly address the challenges of unaligned patient records and temporally irregular observations, they do not account for heterogeneity of progression among patients. Our focus in this paper is on those diseases that exhibit large inter-population variations. For instance, in the case of HD, it is well known that CAG repeat length at conception in the HTT gene (i.e. initial CAG length, or simply CAG length) is associated with the age of disease onset (Ross *et al.* 2014), and the rate of progression. To truly understand progression in the presence of such heterogeneity, statistical models must account for the sources of variation. Our primary contribution in this paper is the development of a CTHMM model whose state transition parameters are conditional on patient characteristics, and we propose an optimization method for model inference. We assume the rates of progression, modeled via a generator matrix in a CTHMM, depend on one or more time-invariant patient characteristics such as gender, ethnicity, genetic traits, etc. We use a log-linear model to encode this dependency, and we use efficient second-order gradient methods to learn the parameters of the log-linear model.

The rest of this paper is organized as follows. Section 2 gives a summary of related work. In Section 3, we describe the model setting, and the proposed optimization method. In Section 4, we present results from applying the proposed method to a real-world observational dataset on Huntington’s disease. Section 5 discusses some of the remaining challenges as well as future research directions.

## 2 Related work

Disease progression models (DPM) provide a basis for learning from prior clinical experience and summarizing knowledge in a quantitative fashion, and have been widely used in medical informatics, e.g. in drug development and analyzing diseases such as Alzheimer’s disease (Saboo *et al.* 2021), dementia (Cho *et al.* 2021), and Parkinson’s disease (Severson *et al.* 2020). Cho *et al.* (2021) use clinical and demographic data (apolipoprotein E4 and sex) to develop a progression model for measuring the AD continuum, whereas Mould *et al.* (2007) use DPMs to design informative clinical trials to detect drug effects. Marchuk *et al.* (2018) proposed a hidden Markov model (HMM) based DPM to measure the circadian cycle of human activity. More recently, a HMM based progression model has been used for modeling the progression of COVID-19 in multiple aspects such as its natural progression (Zhang *et al.* 2021) and spatio-temporal spread in geography (Zhou *et al.* 2021).

Our work belongs to the line of research focusing on utilizing statistical and machine learning techniques in disease progression modeling, based on longitudinal clinical observations. Mor *et al.* (2021) provide a systematic study of different hidden Markov models from a technical viewpoint. Most of these techniques apply probabilistic models where observations are available at discrete and regular intervals. However, for irregular clinical measurements, Wang *et al.* (2014) use a continuous-time progression model (CTHMM) from discrete-time observations to learn the full progression trajectory and applies the model to a Chronic Obstructive Pulmonary Disease cohort. Alaa and van der Schaar (2019) proposed a discrete-space state-space modeling based on deep learning techniques, which can retain long term memory between states by removing the Markovian assumption.

However, the aforementioned studies assume that the transition parameters are shared among the whole population, i.e. rates of progression are homogeneous in the targeted population. Such assumptions rarely hold in practice, particularly for chronic diseases in which the rates of progression could exhibit a significant amount of variation. Only a few efforts relax this assumption and allow the transition rate to vary. Recently, Severson *et al.* (2020) proposed a personalized input-output HMM where each patient is allowed to vary from the population only in the observation model in terms of medication usage. However, this model did not allow variations in transition stages depending on patient’s demographic and genetics data unlike our model. In this article, we propose a CTHMM model with rates of progression that depend on one or more static covariates such as patients’ genetics and demographic data.

## 3 Materials and methods

### 3.1 Model setting

**Figure 1:**
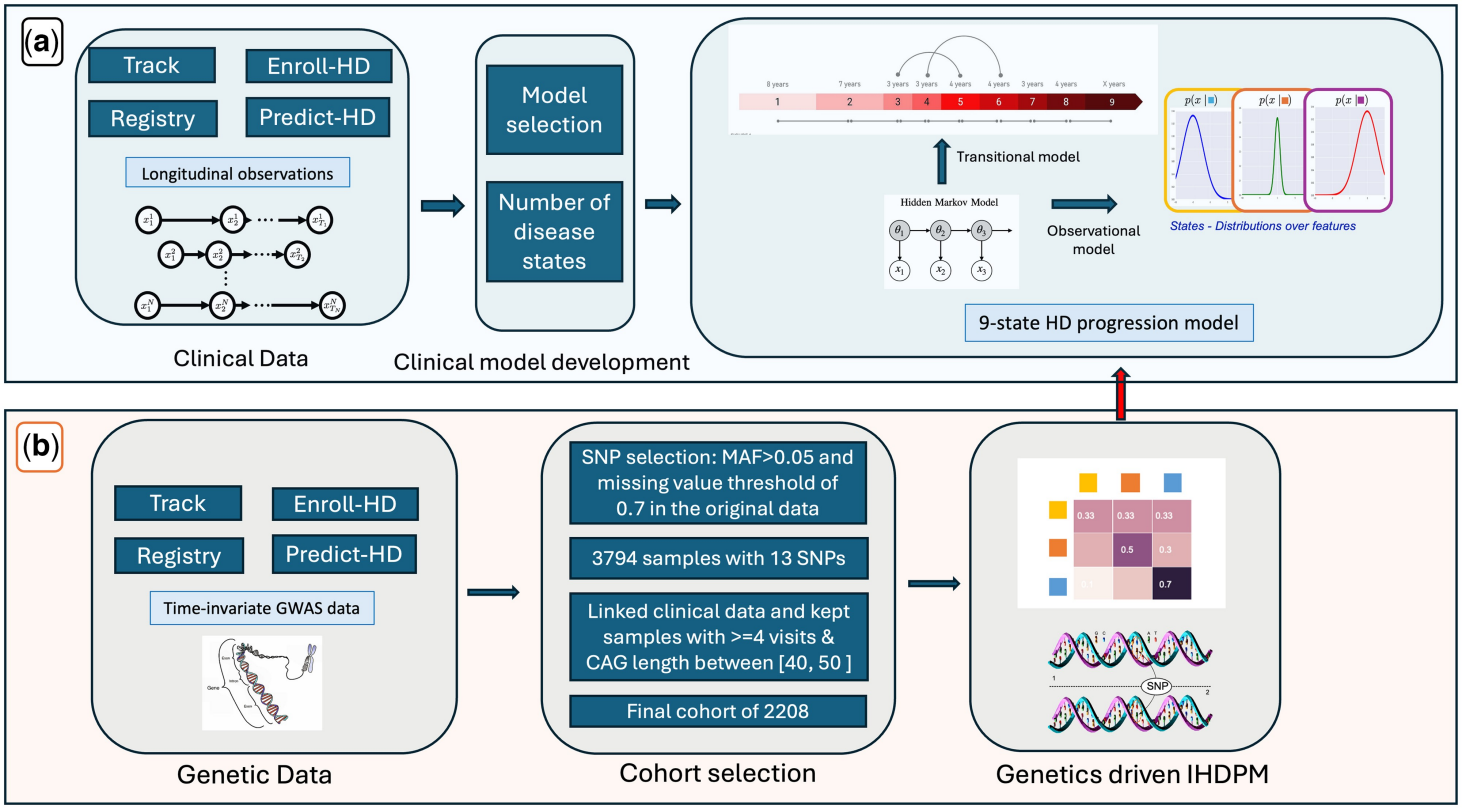
The overall framework for our framework—(a) The top blue panel shows our prior IHDPM model built on clinical data with 9 states. (b) The bottom panel shows steps for utilizing patients genetic characteristics for modeling IHDPM, where patients characteristic determine the transitional model of IHDPM.

We model the progression of a target disease based on longitudinal clinical observations from a cohort of patients who are at risk or have developed the target disease. In a previous study (Sun *et al.* 2019), a probabilistic framework of the disease progression model (IHDPM) (Fig. 1) based on a Continuous Time Hidden Markov Model (CTHMM) was proposed for Huntington’s disease from clinical assessments. In this study, we take advantage of this CTHMM model to discover the impact of genetic makeup on the diverse progression patterns of HD patients. A probabilistic CTHMM model typically consists of two major components. The first component is referred to as the transition model, where a Markov Jump Process is used to model the transition of disease states. The underlying progression is assumed to be continuous time. The second component is referred to as the observation model. The conditional distribution of clinical observations given disease states characterizes the observation model. Changes in the clinical observations manifest the transition of the underlying Markov Jump Process.

**Table 1:** Notations and meanings.

*N*	Number of patients
*M*	Number of progression states
t/τ	Discrete/continuous timestamp
S	Disease states
X	Observed clinical features
*K*	Dimension of clinical features
Z	time-invariate Genetic covariates
*L*	Dimensions of covariates
π	Initial state distribution
Q(β,Z)	Transition generator matrix
β	parameter in transition model
A(δ)	Transition probability matrix over time span δ

In the conventional Markov Jump Process, the transition parameters are assumed to be the same for the whole cohort. Such assumptions may not always hold true in practice, particularly for complex diseases in which the rate of progression may be affected by characteristics of patients. In this study, we relax the assumption and assume that the transitions between disease states depend on some patient genetic characteristics other than the clinical observations (Fig. 1). We also assume that the patient’s genetic characteristics are time-invariant. In the rest of this paper, the patient characteristics under consideration are referred to as covariates.

### 3.2 Notations

We assume that there are *M* different progression states, and *K* different clinical observations which are tracked longitudinally. The clinical features of patient *n* at his *t*th time stamp are denoted as $X_{nt}=\left\{X_{nt1},\ldots ,X_{\mathrm{ntK}}\right\}$. Please refer to (Table 1) for notations.


(1) $$\text{log}\;\left(Q_{ij}\right)=\beta_{i,j}^{⊤}Z,$$

where $\beta_{i,j}$ and Z are *L*-by-1 vectors. Note that adding a bias term on the right-hand side of (1) is equivalent to expanding Z with an additional dimension of constant 1. Therefore, we use the more general form and do not explicitly separate a bias term. We use β to denote the collection of $\beta_{i,j}$ for all i≠j, and refer to β as the covariate parameter.

Although the underlying progression is assumed to be continuous time, we only observe the clinical features X at discrete times. Assume there are *N* patients, and patient *n* has $T_{n}$ longitudinal observations with time stamps $\tau_{1},\ldots ,\tau_{T_{n}}$. Let $S_{m}$ denote the *m*th disease state with m=1,…,M. Let $S_{n,t}$ denote the disease state of patient *n* at his *t*th observation, and $S_{n}=\left\{S_{n,1},\ldots ,S_{n,T_{n}}\right\}$ denote the disease state sequence of the patient.

Given the covariate vector Z, the covariate parameter β, the transition probability from disease state *i* to *j* with a time span δ is defined as the following:


(2) $$\begin{array}{c} A_{ij}(\delta ,Z)=P(S_{t}=j|S_{t-1}=i,\tau_{t}-\tau_{t-1}=\delta ;Q\left(\beta ,Z\right))\\ =\text{expm}{\left(\delta Q\left(\beta ,Z\right)\right)}_{ij}, \end{array}$$

where expm(·) denotes the matrix exponential.

Let P(X|S) denote the conditional distribution of clinical features X given disease state *S*. In the rest of this paper, we refer to conditional distribution as the observational model. The proposed algorithm in this paper works for any observational model in the exponential family. For simplicity of discussion, in this paper, we assume that $X_{k}|S$ for k=1,…,K follow independent Gaussian distributions, i.e. $X_{k}|S=s∼N\left(\mu_{s,k},\sigma_{s,k}^{2}\right)$, where $\mu_{m,k}$ and $\sigma_{m,k}^{2}$ are the mean and variance of the *k*th clinical feature given disease state *m*. The observation model will be referred to as the *Gaussian* observation model in the rest of this paper. We use Θ to denote the collection of parameters in the proposed disease progression model. Note that the underlying disease state sequences S are not observed. The goal is to estimate both Θ and S simultaneously.

### 3.3 Inference

Model inference based on the proposed model is challenging mainly for two reasons. First, although the underlying disease progresses in a continuous time manner, the clinical features are observed at discrete times, and the time stamps of observations could be non-equidistant. Second, the transition generator matrix *Q* depends on genetic covariates Z. In this paper, we perform the Maximum Likelihood Estimate (MLE) and iteratively update Θ and S. We use the Viterbi algorithm (Viterbi 1967) to update the state sequences S, and use the Expectation-Maximization(EM) algorithm (Dempster *et al.* 1977) to perform MLE for estimating Θ.

Let’s assume that there are *N* independent subjects/patients. Both $X_{n}$ and $Z_{n}$ are observed data from patient *n*. Let $S_{n}\left(\tau \right)$ and $S_{n}$ denote the continuous Markov Jump Process and the discrete disease state sequence of patient *n*. Let $D_{n}=(X_{n},Z_{n},S_{n})$ denote the complete data (observed data and underlying disease progression trajectory) from patient *n*. The logarithm of complete likelihood of a patient can be written as follows:


(3) $$\begin{array}{cc} l(\Theta ;S,X,Z)=\text{log}\;P\left(X,Z,S,S(\tau );\Theta \right) & \\ =\text{log}\;P\left(S_{n,1}=s_{n,1}\right) & \\ +\sum_{t=1}^{T_{n}}\;\text{log}\;P\left(X_{n,t}|S_{n,t}\right) & \\ +\text{log}\;P\left(S_{n}|S_{n,1},X_{n},Z_{n}\right). & \end{array}$$

The three terms on the right-hand side are from the initial probability, transition model, and observational model, respectively. Next, we discuss the EM algorithm for estimating the parameters.

*E-Step*. In the E-step, we calculate the expected value of the log complete likelihood with respect to the conditional distribution of latent disease progression process S and S(τ), given observed data X and Z:


(4) $$\begin{array}{c} E_{p(S,S(\tau )|X,Z;\Theta^{\left(t\right)})}[\text{log}\;p(X,Z,S,S(\tau );\Theta )]\\ \;=E_{p(S|X,Z;\Theta^{\left(t\right)})}[\text{log}\;\pi +\text{log}\;p(X|S)]\\ \;+E_{p(S,S(\tau )|X,Z;\Theta^{\left(t\right)})}[\text{log}\;p(S,S(\tau );\Theta )]. \end{array}$$

Given Θ, the observations X, and Z, we obtain the state sequences S by the Viterbi algorithm. Since it is a standard method for obtaining state sequences in Hidden Markov Models, details of the method are skipped in this paper.

*M-Step*. In the M-step, we update the parameters Θ, which include the genetic covariate parameter β, initial probability parameter π, and the parameters in the observation model. Note that if the transition generator matrix *Q* does not depend on genetic covariates, the transition generator matrix *Q* is directly updated instead of β. Analytical solutions for updating *Q* is available. The explicit form can be found in equation (II.8) of Philipp Metzner and Schütte (2007). When elements of *Q* depend on genetic covariates Z, no analytic solution for β is available. In the disease modeling context, the number of disease state *M* usually is small. Therefore, we use the second order method (i.e. Newton-Raphson method) to update β followed by *Q* as per Equation (1).

The initial probability π can be updated by the follows:


(5) $$\pi_{i}=\frac{\sum_{n=1}^{N}P\left(S_{n,0}=i|X,Z,\pi^{\prime },\beta^{\prime }\right)}{\sum_{n=1}^{N}\sum_{j=1}^{M}P\left(S_{n,0}=j|X,Z,\pi^{\prime },\beta^{\prime }\right)}$$

The other parameters that need to be estimated are the parameters of the observational model, i.e. the estimation of Gaussian parameters of each state $\mu_{m,k}$ and ${\hat{\sigma }}_{m,k}$2, which will define the characteristics of clinical observations of each HD stage. Since previous disease progression model by Sun *et al.* (2019) discovered nine distinct stages of HD progression which were also clinically interpreted for their ability to characterize disease heterogeneity, we fixed our observation model to their model for ease of interpretability and then we focus on finding the impact of co-variates on the progression between those nine states. We note that our model is generic enough to re-learn the observational model with different number of disease stages through these E-M algorithms.

The overall flow of the algorithm is summarized in Section 2, available as supplementary data at *Bioinformatics* online.

## 4 Experimental results

### 4.1 Dataset

In this section, we present the results of applying the proposed model to an integrated dataset from four large prospective observational studies of Huntington’s disease(HD) (Ross *et al.* 2014), which are Enroll-HD (Mestre *et al.* 2016), REGISTRY (Orth *et al.* 2011), TRACK-HD/TRACK-ON (Tabrizi *et al.* 2013, Papoutsi *et al.* 2014), and PREDICT-HD (Paulsen *et al.* 2008). The participants in these studies were required to visit the study sites approximately annually and underwent a comprehensive battery of clinical assessments. In this paper, participants with CAG repeat length ≥36 are referred to as the cases as suggested by Walker (2007).

Genome-Wide Association (GWA) data were collected for most of the visits in the integrated dataset and were curated into a combined cohort (GWA12345) in a previous study (Lee *et al.* 2019). We linked the GWA data with the combined clinical data curated for the IHDPM model (Sun *et al.* 2019) to build a combined clinico-genomic model, which led to a total number of 4179 samples who had both genomic data and clinical observations.

In this study, we selected 19 previously validated SNPs that have been reported to have a significant impact on HD onset (Lee *et al.* 2019) as the initial candidate pool of genetic markers. We kept SNPs with minimum allele frequency (MAF) >0.05 and a missing value threshold of 0.7, which resulted in 13 final SNPs with 3794 total samples. The cumulative coverage of the original 19 SNPs is shown in Fig. 3, available as supplementary data at *Bioinformatics* online.

Since longitudinal information is crucial for disease progression modeling, we kept subjects who had at least four temporal observations to build the disease progression model, which otherwise ranges from 1 to 14. Further, we kept subjects with CAG repeats between 40 and 50, which led to the final cohort of 2208 samples.

To tackle outliers, missing values and noise, we first performed a robust Bayesian latent variable analysis (Ghosh *et al.* 2017) and extracted nine latent factors from 44 raw clinical assessments. The latent factors captured heterogeneous disease progression directions in the domains of motor, functional and cognitive manifestations. Thus, the number of clinical features (*K*) in this study equals to 9. Finally, the 9 factors obtained from the 44 pre-filtered clinical observations from the IHDPM study were used for modeling the disease progression model.

### 4.2 Experimental setup

We used the previously defined observational model by Sun *et al.* (2019) in this paper since the disease states discovered by that paper were clinically significant for defining disease characteristics. Specifically, we used the exact 9 states that were discovered in this study for our observational model. We refer to Sun *et al.* (2019) for details of this analysis. Throughout the rest of this section, we set the number of disease states *M* to be 9.

It is known that CTHMM based models can be sensitive to initialization. Since our model assumes that genetics only impact disease progression among the stages of HD rather than affecting the individual disease states directly, we assume that the genetic factor only affects the transition generator matrix. We initialized the parameters of the observational model, namely, μ, σ, π from the previous IHDPM model.

To initialize the parameters of the transitional model of the GWA-enriched IHDPM, we started with multiple random initial values and ran the algorithm for a small number of iterations (e.g. 10). The results with the maximum likelihood were then used as the initial values for building the model.

**Figure 2:**
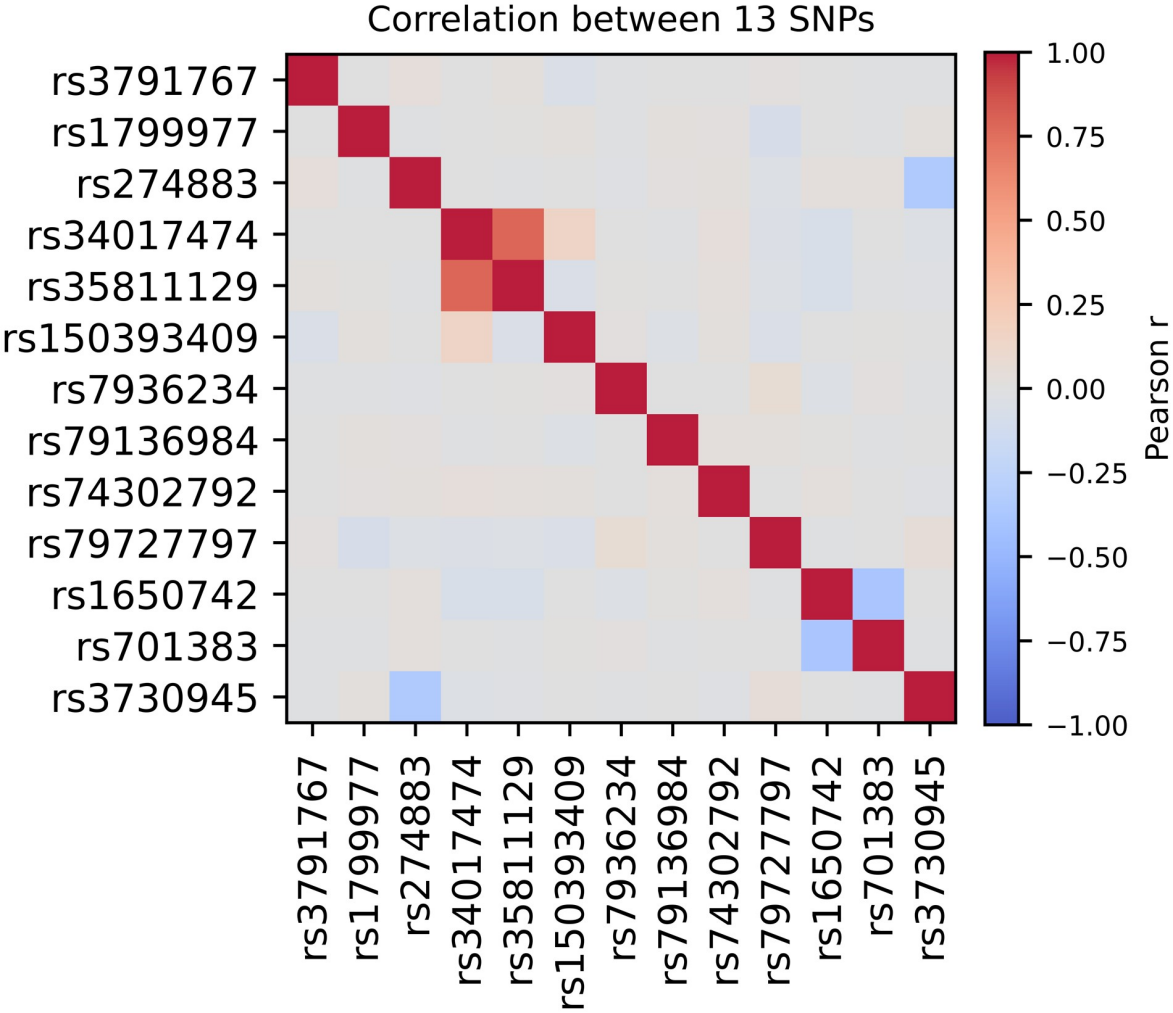
Correlations among the top 13 SNPs.

The initialization of other parameters, such as β, is critical to make sure that the model is not stuck to local minimum of the objective function. We experimented with two ways of initializing the β parameters: with random initialization of β with standard normal and using a two-step approach. In the later approach, we first ran our Genetics-driven IHDPM on each of the SNPs separately to learn their individual effects on disease progression, and then initialized the final β parameters with the individual β of each SNP. Note that the later initialization technique of β parameters is justified based on the observation that the SNPs were mutually independent as shown in Fig. 2. Fig. 1, available as supplementary data at *Bioinformatics* online shows the effect of initialization of the β parameters on model fitness. Since we observed that the initialization based on the individual effects provides better goodness of fit, we present results from this model to analyze the effect of genetic factors on disease progression.

The CAG repeat length in the HTT gene has long been identified as one of the most important genetic factors for HD. It has been well established that the repeat number determines whether a person will or will not develop the disease, as well as the severity of the symptoms. It has been found to be inversely correlated with age of clinical onset (Langbehn *et al.* 2011) and accounts for ∼60% of the variation in age of clinical onset. However, the effect of the CAG repeat length on the rate of disease progression is less clear. To investigate this, we built a CTHMM model with CAG repeat length incorporated as an additional covariate along with genetic factors to check for the combined genetic effect on HD progression. We compare the models with and without CAG length as a covariate. Since our model is unsupervised, we evaluated the obtained model based on the goodness of fit of the obtained model, using log-likelihood (Tan *et al.* 2006) on the held out test set.

Furthermore, we used a bootstrap approach to evaluate the model fitting with 60 random samplings of the original cohort. Within each bootstrap iteration, we randomly split the participants into a training cohort (80%) and a testing cohort (20%) to estimate the model parameters. The baseline model and the CAG model were trained on the training cohort, and the log prediction likelihood values on the testing cohort were calculated. This procedure was repeated 100 times to estimate the statistical significance of the β coefficients.

All experiments were conducted on a Linux-based high-performance computing node (Intel Xeon, 3.0 GHz, 64 GB RAM). Execution time was measured using Python’s time module across ten repeated runs to obtain average and standard deviation β values. The observed runtime increased proportionally to the length of the observation sequence and quadratically with the number of hidden states, in agreement with the theoretical complexity of $O(N^{2}T)$. It took ∼20 minutes to finish one run of complete genetics-aware IHDPM.

### 4.3 Result

The combined genetic model with both CAG length and SNPs had the best model fitness in comparison to the models built on either CAG length or SNPs (the log-likelihood scores of these three models are shown in Fig. 2, available as supplementary data at *Bioinformatics* online). We will use the model that uses both SNPs and CAG as covariates for all further analyses.

In this section, we describe results of our combined progression model built on CAG length and genetics data in multiple aspects: (i) effect of CAG length on the progression model, (ii) effect of SNPs and corresponding genes at state transitions, and (iii) any common progression trajectory of HD based on genetics.

#### 4.3.1 Effect of CAG length

**Figure 3:**
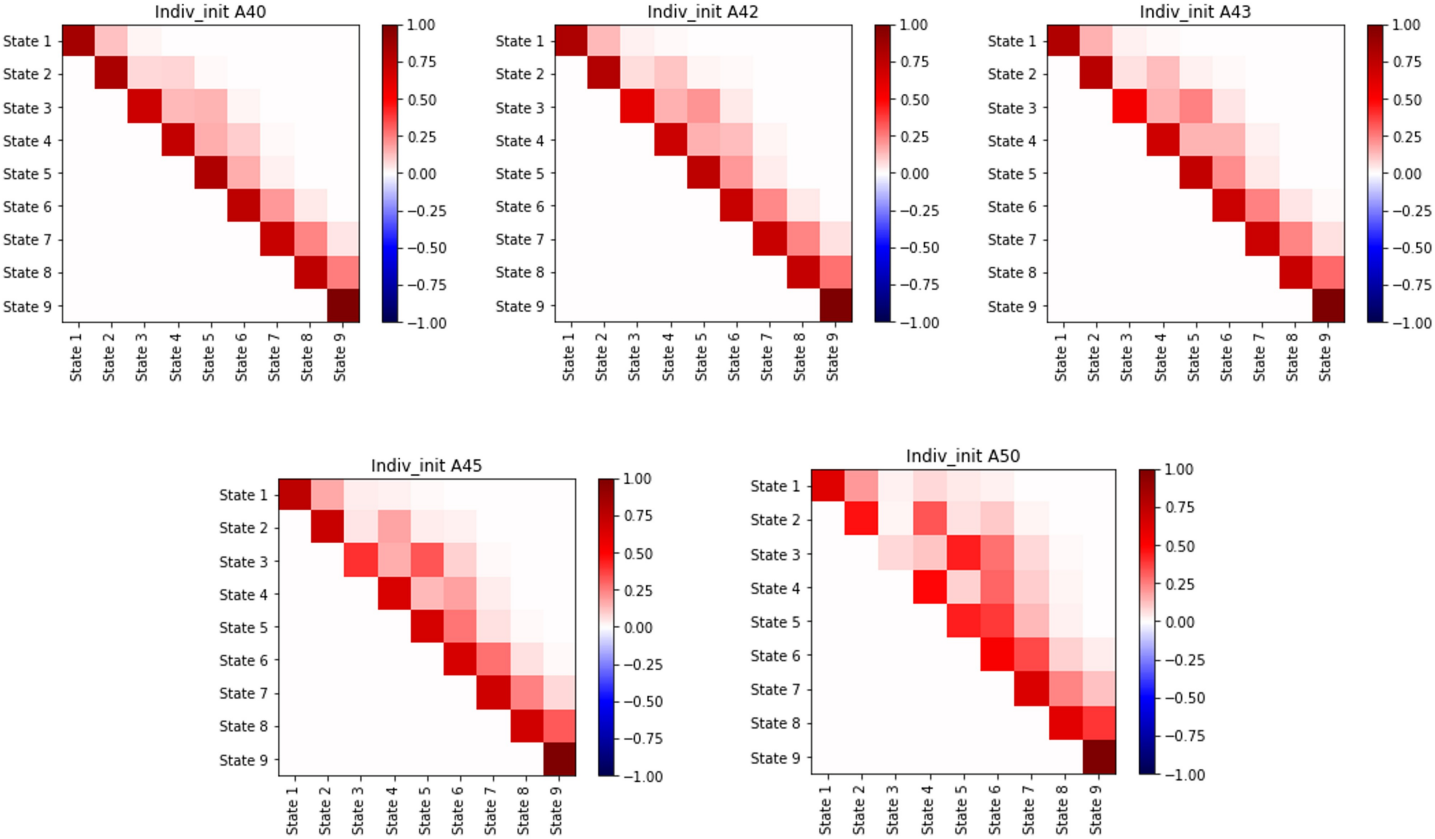
Effect of CAG length on transition generation matrix for δ=1 year. Each cell represents the transitional probability from state *i* (*y*-axis) to state *j* (*x*-axis) of HD within 1 year. For better visualization, probabilities <0.005 were truncated to zero.

Since CAG length has been reported as the topmost contributor in disease progression of HD, we first report the contribution of CAG length on the disease progression. In particular, we explore the details of the differences among the rates of progression for different CAG length groups in our combined model built on CAG length and genetics data. Specifically, we calculated the transition probability matrices with time gap δ equal to 1 year for groups of patients with CAG length with ranges from the value sets {40,42,43,45,50} using the Equation (1). The heatmaps of transition probabilities for 1 year are shown in Fig. 3. For clarity, we show only the cells in which the absolute value of the probabilities is >.005. The diagonal line elements at each panel represent probabilities for staying in each of the nine stages of HD, and the next off-diagonal entries show the jumping probabilities to the next state from each of the first 8 states, and so on. The lower CAG value of 40 has higher probabilities for staying in the same disease state at the end of one year, as compared to the higher CAG values, which have lower probabilities for staying in the same disease state. On the other hand, the jumping probabilities represented by the upper off-diagonal cells are impacted to a greater extent by the higher values of CAG length. For example, jumping probabilities are significant mostly between two adjacent stages for CAG = 40, whereas the jumping probabilities between distant stages that are ≥2 steps away become more significant as the CAG length is increased. This became especially evident for the transitional disease stages (i.e. Stages 4–6). For example, the CAG 50 group have greater probabilities to move to a more advanced disease state at the end of one year at stage 2–4. Such an observation is consistent with the clinical experience that patients with larger CAG length progress faster. The results indicated that rates of disease progression differ with different CAG lengths.

#### 4.3.2 Finding the impact of SNPs and CAG on HD progression

**Figure 4:**
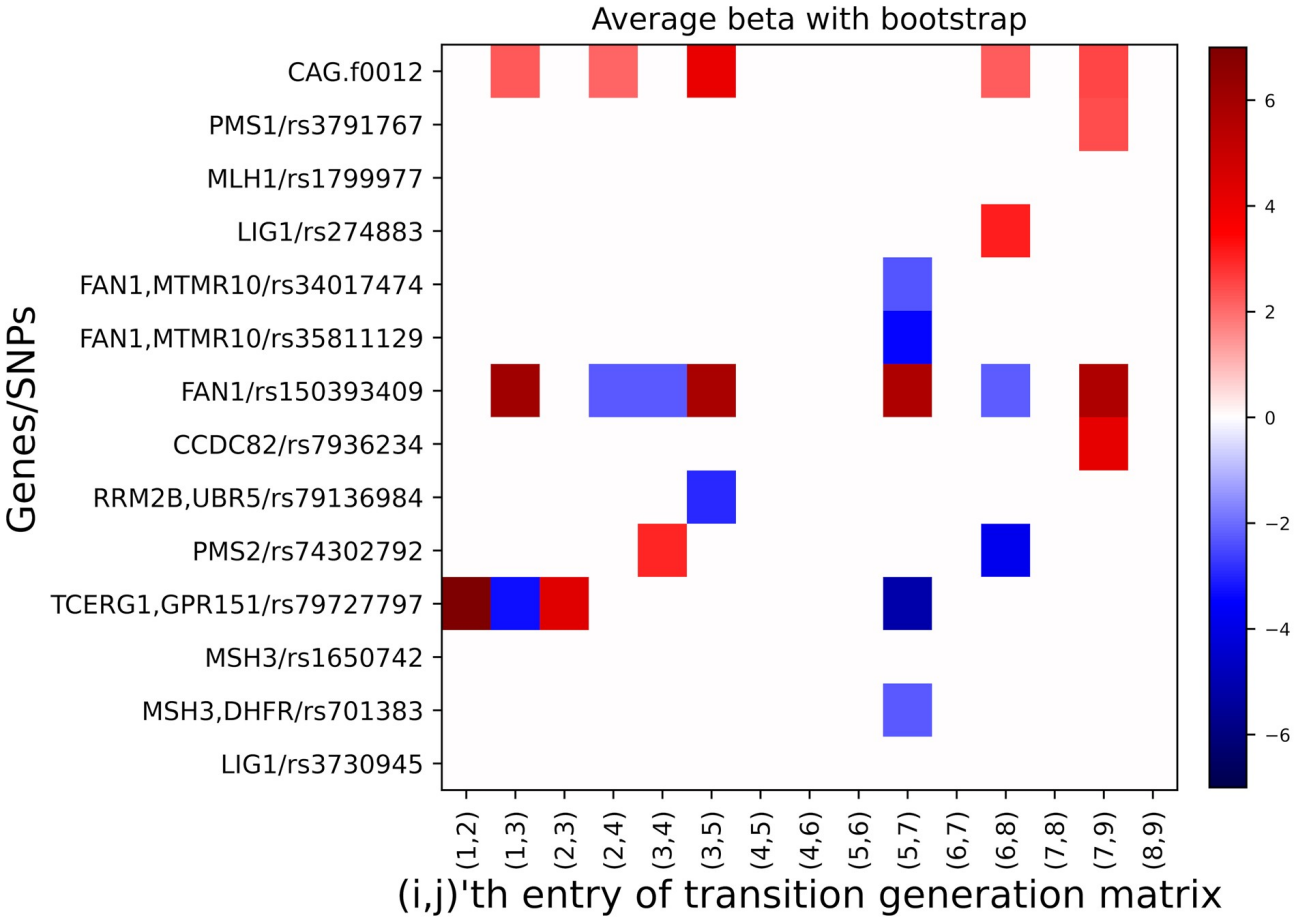
Effect of CAG length and SNPs on HD progression among the 9 different states over 1 year. The *x*-axis represents the 15 possible state transition pairs which are two hops away and *y*-axis represents impact of all genetic factors on the state-transitions. For better visualization, small *beta* coefficients between [−2,2] are not shown.

As a next model, we build a combined progression model taking all genetics factors including both CAG length and SNPs as the covariates affecting disease progression. Furthermore, the maximum number of state transitions between 9 states were experimented as model parameter and the transition to a state which is three states after the current state is negligible in our forward progression model. Therefore, we visualize the β coefficients of the SNPs and CAG length in Fig. 4, where the rows represent genetic factors and the columns represent the transition between two states (i, j), where j≤i+2. Each entry of the matrix represents the genetic impact of SNPs and CAG on the transition from state *i* to *j*. The positive coefficient (represented by red cells of Fig. 4) represents that the corresponding genetic marker accelerates the disease progression while the negative coefficient (represented by blue cells in Fig. 4) represents that the corresponding genetic factor slows down the disease progression.

**Figure 5:**
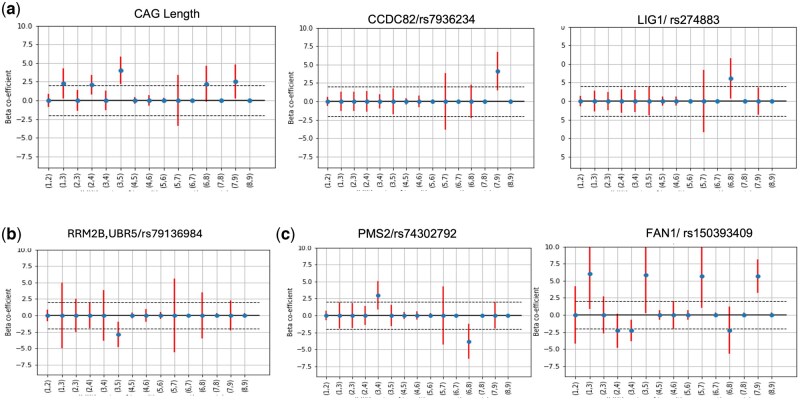
Statistical significance of the impact of CAG length and SNPs on HD progression at different states using 100 bootstrap runs: (a) genetic factors (CAG length, CCDC82 and LIG1) accelerate the disease progression at most of the stages, (b) SNP in RRM2B, UBR5 decelerates the progression and (c) SNPs in PMS2 and FAN1 can both accelerate and decelerate disease progression at different states. In each of these plots, beta-coefficients (dots) are represented along with 95% confidence interval (vertical bars) for the 15 possible transitional pairs between 9 states (*x*-axis).

The results show that CAG length accelerates disease progression at most of the states (e.g. from state 1, 2, 3, 6, and 7), while some SNPs may have both positive and negative impacts at different stages of disease progression. We show the 95% confidence interval of the coefficients for each SNP and CAG length in Fig. 5 as estimated by the bootstrapping experiments. We consider both effect size (β>=2) and confidence interval of each SNP for statistical significance. In these figures, we can observe that CAG length impacts early stages of HD for faster progression, while the impacts on later stages such as 6 and 7 are not statistically significant, as the confidence interval crosses 0. Similarly, CCDC82 and LIG1 impact the disease progression in late stages of 7 and 6, respectively. In contrast, RRM2B has protective impact for disease transitions, i.e. it slows down the disease progression at the transitional stage of 3. On the other hand, PMS2 and FAN1 have both positive and negative impact at different stages of disease progression. This shows that our method cannot only discover both positive and negative impact on HD progression, but also how the impact can vary at different stages of disease progression. Furthermore, our method can also highlight the degree of impact of the same gene at different stages of disease progression.

#### 4.3.3 Finding patterns of genetic impact on HD progression

**Figure 6:**
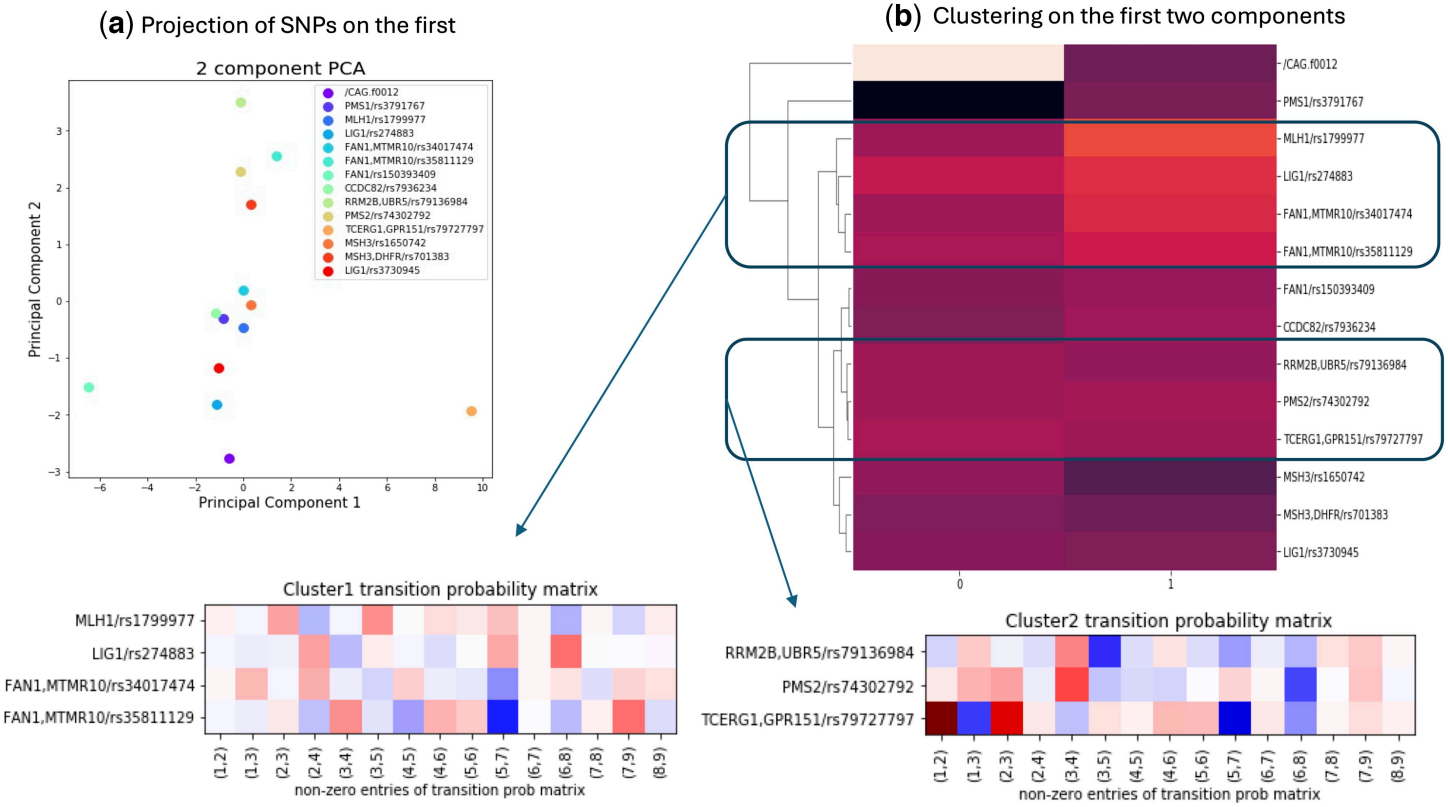
Clusters of impact patterns of genetic factors on HD progression. (a) Projection of β coefficients on lower dimension on the first two principal components, (b) clustering of genetic markers on these two components using agglomerate clustering.

We investigated whether there are any significant coherent patterns of impact of covariates on the transition probabilities using both hierarchical agglomerative clustering and principal component analysis (Tan *et al.* 2006). In particular, we first summarized the β coefficients into lower dimensional spaces, as shown in Fig. 6a using the first two principal components, which accounted for >60% of the variation. Then, we used agglomerative clustering using average grouping on these two principal components to find groups of genes that have similar impact coefficients on HD, as shown in Fig. 6b. We discovered two prominent clusters of SNPs having similar impact on transition probabilities. The first cluster comprised of rs1799977, rs274883, rs34017474, rs35811129 coming from genes MLH1, LIG1 and FAN1, and showed the most coherent pattern of impact on transition probability matrix, as shown in the lower panel of Fig. 6b. The second cluster contained RRM28, PMS2 and GPR51 genes. Prior literature indicated that the first cluster contains genes that are involved in DNA repair activity, which is already known to be associated with HD, which validates the effectiveness of our algorithm in discovering patterns of genes with common disease mechanisms. The second cluster may be a potential new pattern of genetic impact. Such insights have the potential to generate new hypotheses related to possible mechanistic effects of such genes on clinical phenotypes in HD.

## 5 Discussion

Diseases that are known to have highly divergent progression trajectories pose problems for patients, clinicians and researchers, and tools that can help refine current predictions in this setting can represent a significant step forward. A disease such as HD, which may be identified at a very young age, but may not progress to symptom onset for literally decades, can represent a significant burden for patients who must make major life choices in the face of significant uncertainty. For clinicians, it can complicate decisions about when to advise a patient to make lifestyle changes or begin taking medications. For researchers, it can significantly complicate efforts to demonstrate treatment efficacy in a disease modifying agent if tools to predict progression are not sufficiently precise.

Additionally, the heterogeneity of progression to onset clearly points to potential disease modifiers. While such modifiers often relate to co-morbidities, exposures, or overall health status, there is certainly reason to explore and identify relevant genetic variations that may represent contributing factors. In the case of serious, life-altering diseases with no known cure, such research is particularly important.

By identifying previously unidentified impacts of SNPs on the rate of progression, we may help to support decision-making today if clinicians can better counsel patients and make treatment recommendations. What is more important, however, is that these associations may identify mechanisms of action that could lead to better understanding of the factors contributing to variability, and in doing so contribute to the search for improved treatments and preventive measures. Note that this kind of study complements traditional biomarker study by further exploring detailed fine-grained associations between genetic markers and disease progression, which may vary across different stages and to different degrees.

We are encouraged by the fact that some of our associated SNP findings are validated by prior work and relate to DNA repair activity that has been shown to be associated with HD. The immediate next step is to evaluate the new SNPs to determine whether or not there is any reason to believe that the affected SNPs might have an association to a biological process that could account for their impact. Another methodological improvement can be to address the potential correlation between the genetic factors and their temporal associations across states using sparse modeling. Nevertheless, while causation cannot be proven on the basis of this analysis, alone, if the analysis can produce viable candidates for further investigation, it may be an important step in the process toward improved understanding and treatments.

## 6 Conclusion

There are many diseases with established genetic factors, including Huntington’s disease, that are characterized by variable rates of progression, the causes of which are not completely understood. This uncertainty poses challenges to decision making by clinicians and patients, as well as complicating research, particularly with respect to efforts to delay progression. We developed a genetics driven probabilistic disease progression model for HD in order to facilitate identification of genetic factors beyond the huntingtin gene that may have an impact on the progression of HD from an asymptomatic to a manifest state. Multiple clinical observational studies along with a genome wide association (GWA) study were used to build the model. Several SNPs were discovered to have previously undocumented affects at different stages of disease progression for HD. The next step would be to evaluate whether or not there is any reason to believe that the affected SNPs might have an association to a biological process that could account for their impact. This discovery may shed light on the potential mechanistic impact of previously unidentified genes on HD. This result also establishes the potential application of this methodology to other genetic diseases.

## Supplementary Material

btag072_Supplementary_Data

## Software availability

The codebase for IHDPM is available at https://github.com/BiomedSciAI/IHDPM.
