# Supplementary material for: From genes to trajectories: mapping genetic influences on Huntington’s disease progression
Source: Bioinformatics. 2026 Feb 15;42(3):btag072. doi: 10.1093/bioinformatics/btag072 (PMC13003314; doi:10.1093/bioinformatics/btag072)
Supplement: btag072_Supplementary_Data [file btag072_supplementary_data.pdf]

From Genes to Trajectories: Mapping Genetic Influenceson Huntington's Disease Progression

A Preprint

Sanjoy Dey
IBM Research
Yorktown Heights, NY 10598
deysa@us.ibm.com

Zhaonan Sun
IBM Research
Yorktown Heights, NY 10598

John Warner
CHDI Management
CHDI Foundation,
Princeton, NJ

Eileen Koski
IBM Research
Yorktown Heights, NY 10598

Elif Eyigoz
IBM Research
Yorktown Heights, NY 10598

Swati Sathe
CHDI Management
CHDI Foundation,
Princeton, NJ

Cristina Sampaio
CHDI Management
CHDI Foundation,
Princeton, NJ

Jianying Hu
IBM Research
Yorktown Heights, NY 10598

January 14, 2026

Supplemental Section

1 Figures

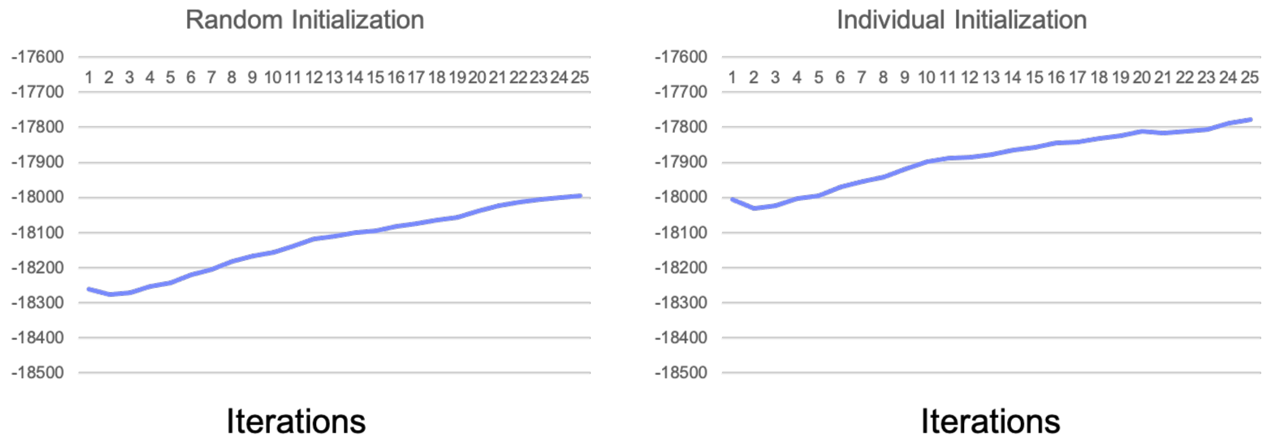

Figure 1: Log likelihood of two best models with two initialization techniques.

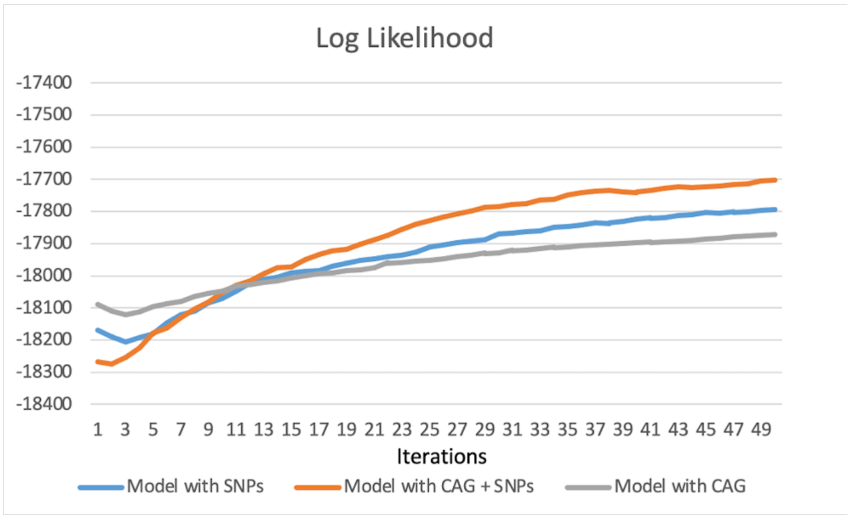

Figure 2: Model comparisons based on goodness-of-fit of three Genetics-enriched models: 1) using the SNPs as co-variates, 2) using CAG length as a co-variate and 3) both CAG and SNPs as co-variates.

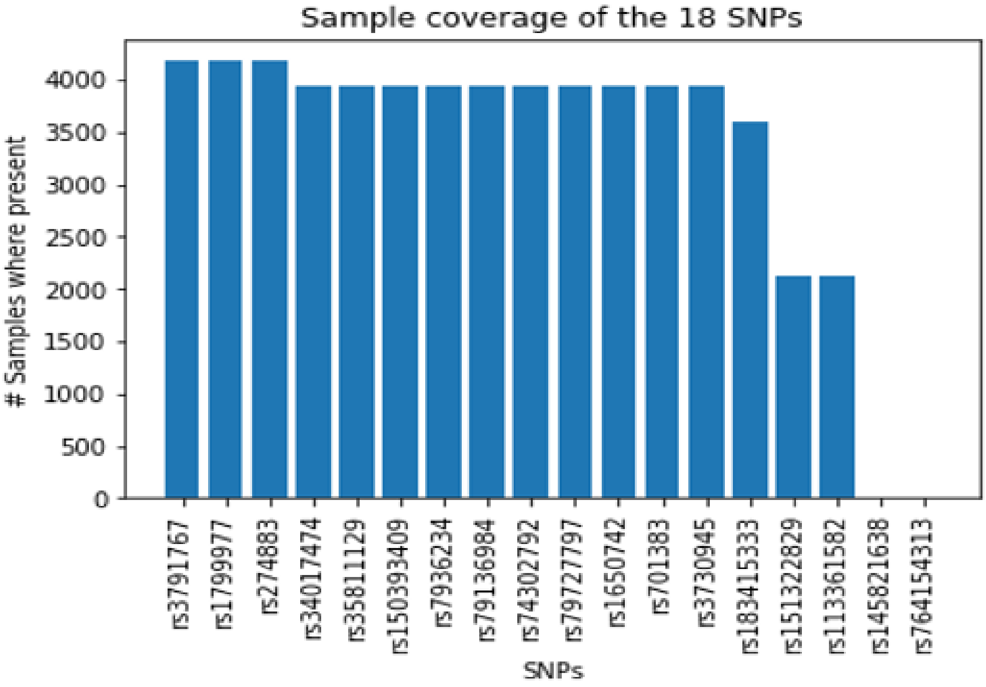

Figure 3: Cumulative Sample coverage of top 18 SNP

2 Algorithm

We assume that there are M different progression states, and K different clinical observations which are tracked longitudinally. The clinical features of patient n at his t-th time stamp are denoted as $\mathbf{X}_{nt} = \{X_{nt1}, \ldots, X_{ntK}\}$. The underlying patient state is assumed to evolve according to a continuous-time Markov process, denoted as $S(\tau)$. The continuous-time Markov process is parameterized by an $M \times M$ transition generator matrix Q, and an $M \times 1$ initial state probability π. Furthermore, we assume that elements of the transition generator matrix depend on one or more static patient characteristics, such as gender, age at baseline, or genetic traits. Throughout the rest of this paper, we refer to the genetic factors as the covariates and denote the vector of genetic covariates as $\mathbf{Z}$. The dimension of the

genetic covariates is denoted as L. Let Q_{ij} denote the (i, j)-th element of the transition generator matrix. We assume that Q_{ij} is a function of the vector of genetic covariates, *i.e.* $Q_{ij} = f(\boldsymbol{\beta}, \mathbf{Z})$. Specifically in this article, we assume the following form of dependence:

$$\log(Q_{ij}) = \beta_{i,j}^{\top}\mathbf{Z},$$

where $\beta_{i,j}$ and $\mathbf{Z}$ are L-by-1 vectors. Note that adding a bias term on the right-hand side of (2) is equivalent to expanding $\mathbf{Z}$ with an additional dimension of constant 1. Therefore we use the more general form and do not explicitly separate a bias term. We use $\boldsymbol{\beta}$ to denote the collection of $\beta_{i,j}$ for all $i \neq j$, and refer to $\boldsymbol{\beta}$ as the covariate parameter.

Given the covariate vector $\mathbf{Z}$, the covariate parameter $\boldsymbol{\beta}$, the continuous-time transition probability matrix A from disease state i to j with a time span δ is defined as the following:

$$\begin{aligned} A_{ij}(\delta, \mathbf{Z}) &= P(S_t = j | S_{t-1} = i, \tau_t - \tau_{t-1} = \delta; Q(\boldsymbol{\beta}, \mathbf{Z})) \\ &= \text{expm}(\delta Q(\boldsymbol{\beta}, \mathbf{Z}))_{ij}, \end{aligned} \tag{1}$$

where $\text{expm}(\cdot)$ denotes the matrix exponential.

Although the underlying progression is assumed to be continuous-time, we only observe the clinical features $\mathbf{X}$ at discrete times. Assume there are N patients, and patient n has T_n longitudinal observations, with time stamps $\tau_1, \ldots, \tau_{T_n}$. Let S_m denote the m-th disease state with $m = 1, \ldots, M$. Let $S_{n,t}$ denote the disease state of patient n at his t-th observation, and $\mathbf{S}_n = \{S_{n,1}, \ldots, S_{n,T_n}\}$ denote the disease state sequence of the patient.

Let $P(\mathbf{X}|S)$ denote the conditional distribution of clinical features $\mathbf{X}$ given disease state S. In the rest of this paper, we refer to the conditional distribution as the observational model. The proposed algorithm in this paper works for any observational model in the exponential family. For simplicity of discussion, in this paper, we assume that $X_k|S$ for $k = 1, \ldots, K$ follow independent Gaussian distributions, *i.e.*, $X_k|S = s \sim N\left(\mu_{s,k}, \sigma_{s,k}^2\right)$, where $\mu_{m,k}$ and $\sigma_{m,k}^2$ are the mean and variance of the k-th clinical feature given disease state m. In this study, we impose a Beta prior on p_{sk}, *i.e.* $p_{sk} \sim \boldsymbol{\beta}(\alpha_0, \beta_0)$. The observation model will be referred to as the *Gaussian* observation model in the rest of this paper. We use Θ to denote the collection of parameters in the proposed disease progression model. Note that the underlying disease state sequences $\mathbf{S}$ are not observed. The goal is to estimate both Θ and $\mathbf{S}$ simultaneously.

2.1 Inference

Model inference based on the proposed model is challenging mainly due to two reasons. First, although the underlying disease progresses in a continuous time manner, the clinical features are observed at discrete times, and the time stamps of observations could be non-equidistant. In this article, we perform the Maximum Likelihood Estimate (MLE) and iteratively update Θ and $\mathbf{S}$. We use the Viterbi algorithm (**?**) to update the state sequences $\mathbf{S}$, and use Expectation-Maximization(EM) algorithm (**?**) to perform MLE for estimating Θ.

Assuming that there are N independent subjects/patients. Both X_n and Z_n are observed data from patient n. Let $\mathbf{S}_n(\tau)$ and $\mathbf{S}_n$ denote the continuous Markov Jump Process and the discrete disease state sequence of patient n. Let $\mathbf{D}_n = (X_n, Z_n, S_n)$ denote the complete data (observed data and underlying disease progression trajectory) from patient n. The logarithm of complete likelihood of a patient can be written as follows:

$$\begin{aligned} l(\Theta; \mathbf{S}, \mathbf{X}, \mathbf{Z}) &= \log P(\mathbf{X}, \mathbf{Z}, \mathbf{S}, \mathbf{S}(\tau); \Theta) \\ &= \log P(S_{n,1} = s_{n,1}) + \sum_{t=1}^{T_n} [\log P(X_{n,t}|S_{n,t}) + \log P(\mathbf{S}_n|S_{n,1}, X_n, Z_n)]. \end{aligned} \tag{2}$$

The three terms on the right-hand side are from the initial probability, transition model, and observational model, respectively. Next we discuss the EM algorithm for estimating the parameters. The first term on the right-hand side can be written as $\log \pi_{s_{g,1}}$, the second term can be derived from the observational model, and the third term on the right-hand side of (2) denoting the transition probabilities can be written as the follows

$$\begin{aligned} & P(\mathbf{S}_g|S_{g,1}, X_g, Z_g) = \\ & \sum_{i=1,j\neq i}^{M} n_T(i,j)\beta_{i,j}^{\top} Z_g - \sum_{i=1,j\neq i}^{M} r_T(i) \exp\left(\beta_{i,j}^{\top} Z_g\right), \end{aligned} \tag{3}$$

where $n_T(i, j)$ is a scalar representing the number of counts of transitions from state i to state j, and $r_T(i)$ is a scalar representing the total time duration that patient g spent in state i. Next we discuss the EM algorithm for estimating the parameters.

E-Step. In the E-step, we calculate the expected value of the log complete likelihood with respect to the conditional distribution of latent disease progression process $\mathbf{S}$ and $\mathbf{S}(\tau)$, given observed data $\mathbf{X}$ and $\mathbf{Z}$: *i.e.* $E_{P(\mathbf{S},\mathbf{S}(\tau)|\mathbf{X},\mathbf{Z},\Theta^{(t)})}\left[l(\Theta;\mathbf{S},\mathbf{X},\mathbf{Z})\right]$.

$$\begin{aligned}\mathbb{E}_{p(\mathbf{S},\mathbf{S}(\tau)|\mathbf{X},\mathbf{Z};\Theta^{(t)})}[\log p(\mathbf{X},\mathbf{Z},\mathbf{S},\mathbf{S}(\tau);\Theta)] = {} & \mathbb{E}_{p(\mathbf{S}|\mathbf{X},\mathbf{Z};\Theta^{(t)})}[\log\pi + \log p(\mathbf{X}|\mathbf{S})] \\ & + \mathbb{E}_{p(\mathbf{S},\mathbf{S}(\tau)|\mathbf{X},\mathbf{Z};\Theta^{(t)})}[\log p(\mathbf{S},\mathbf{S}(\tau);\Theta)].\end{aligned} \tag{4}$$

Note that the first term on the right-hand side of (4) does not contain the covariate parameter $\boldsymbol{\beta}$ since we did not modify the observational model from **?**, and only the second term involves $\boldsymbol{\beta}$. For notational simplicity, we use l_{trans} to denote the second term. In the absence of covariates $\mathbf{Z}$ in the transition model, the explicit form of l_{trans} can be found in (**?**). When elements of the transition generator matrix Q depend on covariates, following a similar derivation, the second term in (4) can be written as follows:

$$\begin{aligned} l_{trans} = \mathbb{E}_{p(\mathbf{S},\mathbf{S}(\tau)|\mathbf{X},\mathbf{Z};\Theta^{(t)})}[\log p(\mathbf{S},\mathbf{S}(\tau);\boldsymbol{\beta}^{(t)})] = \sum_{\delta}\sum_{i,j\in[M]} C_{ij}(\delta) \\ \left(\sum_{k,l\in[M],k\neq l}(\beta_{k,l}Z)\mathbb{E}[N_{kl}(\delta)|\mathbf{S};\boldsymbol{\beta}^{(t)}] - \exp(\beta_{k,l}Z)\mathbb{E}[R_k(\delta)|\mathbf{S};\boldsymbol{\beta}^{(t)}]\right).\end{aligned} \tag{5}$$

In equation (5), $N_{ij}(\delta)$ is a scalar representing the counts of transitions from state i to state j over the time duration δ, $R_i(\delta)$ is a scalar representing the total time that patient spent in state i over the duration of δ.

The term $C_{ij}(\delta)$ is the expected number of transitions from state i to state j over the duration of δ. The expectation of $C_{ij}(\delta)$ can obtained by the second order posterior probability, which is defined as follows

$$\xi_t(i,j) = P\left(S_{n,t-1}=i, S_{n,t}=j|\mathbf{X}_n,\mathbf{Z}_n,\Theta\right). \tag{6}$$

Equation (6) can be obtained by the Baum-Welch algorithm based on the forward-backward lattices and Gibbs sampling are similar to **?**. Since it is a standard algorithm, we will omit the details in this article.

In the following, we describe how to estimate the two auxiliary variables $N_{ij}(\delta)$ and $R_i(\delta)$.

Let $\mathbf{Z}_1,\ldots,\mathbf{Z}_E$ be the unique combinations of covariates values among all subjects $1,\ldots,N$, and $\mathcal{N}_e$ be the set of participants whose covariate value equals to $\mathbf{Z}_e$, and Δ_e be the set of unique time gaps between consecutive observations for subjects g in $\mathcal{N}_e$, for $e=1,\ldots,E$. Equation (5) then can be written as follows:

$$\begin{aligned} l_{trans} &= \sum_{e=1}^{E}\sum_{\delta\in\Delta_e}\sum_{i,j\in[M]}\sum_{k,l\in[M],k\neq l} C_{ij}(\delta)\left((\beta_{k,l}Z_e)\mathbb{E}[N_{kl}(\delta)|\mathbf{S};\boldsymbol{\beta}^{(t)}] - \exp(\beta_{k,l}Z_e)\mathbb{E}[R_k(\delta)|\mathbf{S};\boldsymbol{\beta}^{(t)}]\right) \\ &= \sum_{e=1}^{E}\sum_{\delta\in\Delta_e}\sum_{i,j\in[M]}\sum_{k,l\in[M],k\neq l}(f(\boldsymbol{\beta}_{k,l}) + g(\beta_{k,l}))\end{aligned}. \tag{7}$$

There are multiple methods to compute the two conditional expectation terms $\mathbb{E}[N_{kl}(\delta)|\mathbf{S};\boldsymbol{\beta}^{(t)}]$ and $\mathbb{E}[R_k(\delta)|\mathbf{S};\boldsymbol{\beta}^{(t)}]$. Note that both of these conditional expectations depend on the transitional probability generation matrix Q. Let $Q\left(\boldsymbol{\beta}^{(t)},\mathbf{Z}\right)$ denote the transition generator matrix based on the co-variates $\mathbf{Z}$ and the parameter $\beta^{(t)}$.

Specifically, the conditional expectations can be computed by leveraging a classic method of Van Loan **?** for computing integrals of matrix exponentials. In this approach, an auxiliary matrix H is constructed as $H = \begin{bmatrix} Q & B \\ 0 & Q\end{bmatrix}$, where B is a matrix with identical dimensions to Q. It is shown in **?** that $\int_0^{\delta}\text{expm}(Qx)B\text{expm}(Q(\delta-x))\,\mathrm{d}x = (\text{expm}(H\delta))_{(1:M),(M+1):(2M)}$.

Now, we define two auxiliary matrices for computing $\mathbb{E}[R_k(\delta)|\mathbf{S};\boldsymbol{\beta}^{(t)}]$ and $\mathbb{E}[N_{kl}(\delta)|\mathbf{S};\boldsymbol{\beta}^{(t)}]$. $H_{(i,i)}$ along with $B=I(i,i)$, and $H_{(i,j)}$ along with $B=I(i,j)$ are used for these two computations respectively. $I(i,i)$ is the matrix with a 1 in the (i,i)-th entry and 0 otherwise, and $I(i,j)$ is the matrix with a 1 in the (i,j)-th entry and 0 otherwise. The details of the *expm* method are summarized in Algorithm 1.

Given Θ, the observations $\mathbf{X}$, and $\mathbf{Z}$, we obtain the state sequences $\mathbf{S}$ by the Viterbi algorithm. Since it is a standard method for obtaining the state sequences in Hidden Markov Models, details of the method is skipped in this article.

M-Step. In the M-step, we update the parameters Θ, which include the genetic covariate parameter $\boldsymbol{\beta}$, initial probability parameter π, and the parameters in the observation model. Note that if the transition generator matrix Q does not depend on genetic covariates, the transition generator matrix Q is directly updated instead of $\boldsymbol{\beta}$. Analytical solution for updating Q is available. The explicit form can be found in equation (II.8) of (**?**). When elements of Q depend on genetic covariates $\mathbf{Z}$, no analytic solution for $\boldsymbol{\beta}$ is available. In the disease modeling context, the number of disease state M usually is small. Therefore we use the second order method (i.e. Newton-Raphson method) to update $\boldsymbol{\beta}$.

Next we update the parameters in the observation model. Under the Gaussian model, the parameters such as the initial probability π, μ, and Σ are updated similar to **?**. The overall algorithm is given in Algorithm 2.

for *each state i in S* **do**
  for $\delta = 1$ **to** r **do**

$$D_{(i)} = \frac{\text{expm}(\delta_\Delta H)_{(1:M),(M+1):(2M)}}{A_{kl}(\delta_\Delta)}, \quad \text{where } H = \begin{bmatrix} Q & I(i,i) \\ 0 & Q \end{bmatrix}, \quad E_{\mathbf{S}(\tau)}\left[R_i(\delta)\,|S(\delta) = l, S(0) = k; Q'\right] = D_{(i),k,l} \tag{8}$$

  end
end
for *each link (i,j) in L* **do**
  for $\delta = 1$ **to** r **do**

$$D_{(i,j)} = \frac{\text{expm}(\delta H)_{(1:M),(M+1):(2M)}}{A_{kl}(\delta)} \quad \text{where } H = \begin{bmatrix} Q & I(i,j) \\ 0 & Q \end{bmatrix}, \quad E_{\mathbf{S}(\tau)}\left[N_{ij}(\delta)\,|S(\delta) = l, S(0) = k; Q'\right] = Q_{ij} D_{(i,j),k,l} \tag{9}$$

  end
end

Algorithm 1: expm Algorithm

Input: Clinical features $\mathbf{X}$, time invariate covariates $\mathbf{Z}$, step size $\gamma \in (0,1)$
Output: Covariate parameter $\boldsymbol{\beta}$, initial state probability π, observation parameters $\boldsymbol{\mu}$ and $\boldsymbol{\sigma}^2$, and state sequences $\mathbf{S}$
  Initialize $\mathbf{S}, \pi, \boldsymbol{\beta}, \boldsymbol{\mu}, \boldsymbol{\sigma}^2$
repeat
  `// E-step`
  Update forward and backward lattices $\alpha_s(t)$ and $\beta_s(t)$;
  Update posterior distribution terms: $\gamma_{s_t}(t) = P(S_t = s_t|\mathbf{X}, \mathbf{Z}, \pi, \boldsymbol{\beta})$ and $\xi_t(i,j) = P\left(S_{n,t-1} = i, S_{n,t} = j|\mathbf{X}, \mathbf{Z}, \Theta\right)$;
  Update $\mathbf{S}$;
  Compute $C_{ij}(\delta) = \sum_{n=1}^{N} \sum_{t=1}^{T_n} p(S_{n,t-1} = i, S_{n,t} = j|\mathbf{X}, \mathbf{Z}; \Theta) \mathbf{1}_{\tau_t - \tau_{t-1} = \delta}, \forall \delta \in \Delta$;
  `// M-step`
  Compute the conditional expectation terms $\mathbb{E}[\mathcal{N}_{ij}(\Delta)|k, l, \boldsymbol{\beta}]$ and $\mathbb{E}[\mathcal{R}_i(\delta)|k, l, \boldsymbol{\beta}]$ from the *expm* method;
  Compute $d\beta_{k,l}$ and $H_{k,l}$;
  Update $\boldsymbol{\beta}$ by $\beta_{k,l}^{(t+1)} = \beta_{k,l}^{(t)} - \gamma H_{k,l}^{(t)} d\beta_{k,l}^{(t)}$ for $\forall k \neq l$;
  Update $\pi_i \leftarrow \frac{\sum_{n=1}^{N} p(S_{n,0}=i|\mathbf{X},\mathbf{Z};\Theta)}{\sum_{n=1}^{N}\sum_{s=1}^{M} p(S_{n,0}=s|\mathbf{X},\mathbf{Z};\Theta)}$;
  Update parameters in observation model according to **?**;
until *Convergence*;

Algorithm 2: Optimization Algorithm
